# Evaluation of Almond Shell Activated Carbon for Dye (Methylene Blue and Malachite Green) Removal by Experimental and Simulation Studies

**DOI:** 10.3390/ma17246077

**Published:** 2024-12-12

**Authors:** Adrián Rial, Catarina Helena Pimentel, Diego Gómez-Díaz, María Sonia Freire, Julia González-Álvarez

**Affiliations:** 1 Department of Chemical Engineering, School of Engineering, Universidade de Santiago de Compostela, Rúa Lope Gómez de Marzoa S/N, 15782 Santiago de Compostela, Spain; rialpomares13@gmail.com (A.R.); catarinahelenada.silveiramirandaguedes@usc.es (C.H.P.); mariasonia.freire@usc.es (M.S.F.); julia.gonzalez@usc.es (J.G.-Á.); 2 Center for Research in Biological Chemistry and Molecular Materials (CIQUS), Universidade de Santiago de Compostela, Rúa Jenaro de La Fuente S/N, 15782 Santiago de Compostela, Spain

**Keywords:** activated carbon, almond shell, malachite green, methylene blue, adsorption, breakthrough curve

## Abstract

The present work analyzes the behavior of an activated carbon fabricated from almond shells for the removal of cationic dyes (methylene blue, MB, and malachite green, MG) by adsorption from aqueous solutions. The carbonized precursor was activated with KOH at a 1:2 (*w*/*w*) ratio with the objective of increasing both the surface area and the pore volume. Both non-activated and activated carbon were characterized in different aspects of interest in dye adsorption studies (surface structure, point of zero charge, specific surface area, and pore size distribution). The effect of the dye’s initial concentration and adsorbent dosage on dye removal efficiency and carbon adsorption capacity was studied. Adsorption kinetics were analyzed under different experimental conditions, and different models were assayed to determine the adsorption mechanism. Dye adsorption in the adsorbent surface could be considered the rate-limiting step. Different adsorption equilibrium models were evaluated to fit the experimental data. This adsorbent allowed us to reach high Langmuir adsorption capacity for both dyes (MB: 341 mg·g^−1^, MG: 364 mg·g^−1^ at 25 °C and 0.5 g·L^−1^). Moreover, kinetic and equilibrium adsorption data have been used to simulate breakthrough curves in a packed-bed column using different conditions (bed length, liquid flowrate, and dye initial concentration). The simulation results showed that almond shell activated carbon is a suitable adsorbent for methylene blue and malachite green removal from wastewater.

## 1. Introduction

The presence of pollutants in wastewater continues to be one of the main problems associated to industrial processes (i.e., pesticides, heavy metals, dyes, etc.) [1]. Although the presence of emerging contaminants has increased concern about this problem, other more common contaminants (e.g., industrial dyes) continue to be the target of the development, improvement, and application of new separation operations [2].

Water contamination due to the presence of dyes continues to be a major problem due to the significant losses that are produced (close to 20%) in the industrial processes in which they are used [3]. These losses generate high flowrates of wastewater that increase the cost associated with downstream treatments. In addition to this highly negative fact, the concern is increased due to the low dye concentration (in the order of mg·L^−1^) along with the difficulty of the recovery processes and the costs associated with these operations. Also, the importance of a correct recovery process is justified by the undesirable effects of this type of substance (carcinogenic, mutagenic, teratogenic…) in relation to human health.

In recent years, advanced oxidation processes have been shown to be very effective techniques for the elimination of this type of substance; although, high pollutant concentration tends to favor the overall process. In this way, a previous stage that allows increasing the dye concentration can improve the overall removal performance, as well as reduce the costs associated with the separation process. Thus, the adsorption of dyes in porous materials is considered a suitable solution for this purpose [4]. Different investigations have even indicated that the in situ degradation of previously adsorbed pollutants could avoid the desorption stages that can show some difficulties and reduce the overall yield of the process [5,6]. A significant number of research papers are focused on batch studies, which are essential to obtain valuable information regarding the adsorbent characteristics in relation to the adsorption of a target adsorbate. In any case, the information provided by dynamic adsorption studies using packed bed columns allows for a realistic simulation of the process on an industrial scale [7,8] and to evaluate the specific characteristics of the industrial equipment, mainly of that related with the mass transfer processes. Therefore, it is necessary to carry out an overall evaluation of both the batch and steady-state results, which can provide a more suitable point of view of the adsorption process.

Focusing interest on reducing the costs associated with the overall process of dye recovery by adsorption, as well as on attaining some of the Sustainable Development Goals (e.g., 6—clean water and sanitation and/or 11—sustainable cities and communities), the present work proposes the use of an activated carbon produced from an industrial biomass waste, almond shells, which are available in high quantities, as dye adsorbent [9]. The activation process as well as the activating agent used are important aspects that can affect the adsorption behavior of the adsorbate. Consequently, the degree of carbon microporosity must be enhanced or reduced depending on the adsorbate or other compounds that are present in the wastewater. Previous studies [10] have shown that physical or chemical activation with certain agents (i.e., ZnCl_2_) tend to increase slightly both the specific surface area and pore volume. However, the use of KOH can produce generally important increases in both parameters [11]. Therefore, this agent has been selected to prepare an almond shell activated carbon, also considering the relatively large molecular size of the cationic dyes (blue methylene and green malachite) to be adsorbed. Batch experiments were performed to evaluate the influence of operational conditions (initial concentration, contact time, and carbon dosage), and adsorption kinetics and equilibrium were analyzed to determine the dye adsorption mechanism. The novelty of this work is focused on the one hand on the use of a biomass waste to produce an adsorbent than can enhance dye adsorption and on the other on the use of experimental data at the lab scale to simulate breakthrough curves that allow for the evaluation of the performance of this adsorbent in a large-scale process.

## 2. Materials and Methods

### 2.1. Materials and Chemicals

Milled almond shell was supplied by Biogramasa company (Santa Fe, Spain), and a particle size lower than 5 mm was used as the activated carbon precursor. The activation step was carried out with potassium hydroxide (KOH 85%, Quality Chemicals, Esparreguera, Spain) as the activating agent. Sodium chloride (NaCl, 99.5%, Probus), hydrochloric acid (HCl 37%, Sigma Aldrich, St. Louis, MO, USA), and sodium hydroxide (NaOH 98%, Sigma Aldrich) were used for the point of zero charge determination, and the acid was also used to wash the fabricated carbon. Methylene blue (MB > 83%) and malachite green (MG > 93%) dyes were supplied by Panreac Applichem (Darmstadt, Germany).

### 2.2. Adsorbent Fabrication

Almond shell was carbonized at 873 K for 1 h under nitrogen (34 L h^−1^) in an oven (Nabertherm GmbH, Lilienthal, Germany) by using a temperature ramp of 5 K min^−1^. An inertization step was carried out for 30 min. Carbon activation consisted of a thermal treatment at 1123 K for 2 h of a mixture of carbonized material and KOH using a ratio of 1:2 (*w*/*w*). The temperature ramp and nitrogen flowrate applied were the same as in the carbonization stage. The activated carbon was washed successively with 0.1 M HCl and distilled water and dried overnight at 378 K.

### 2.3. Adsorbent Characterization

The point of zero charge (pH_pzc_) was determined as the pH at which the difference between the final and initial pH was zero. Several flasks were prepared with aqueous solutions (10 mL) of 0.01 M NaCl at several pH values adding NaOH and/or HCl. After pH adjustment, carbon was added (10 mg) to each flask and shaken at 350 rpm and 25 °C for 48 h to reach the equilibrium [12].

Surface area and pore size distribution data were obtained using an ASAP 2020 sorption analyzer (Micromeritics, Norcross, GA, USA). Samples were previously outgassed under vacuum at 573 K. This characterization study was carried out with nitrogen adsorption and desorption data at 77 K and carbon dioxide adsorption data at 298.15 K. The BET equation was used to determine the surface area corresponding to N_2_ and CO_2_ adsorption data. The nitrogen amount adsorbed at a relative pressure of 0.99 was used to calculate the total pore volume. Pore size distribution was determined using the two-dimensional nonlocal density functional theory (2D-NLDFT) model that combines CO_2_ and N_2_ experimental data.

Fourier transform infrared (FTIR) spectra (Varian FTIR 670 spectrometer, Agilent, Santa Clara, CA, USA) were obtained by the preparation of KBr pellets (0.4 mg of carbon with 600 mg of KBr). The wavenumber range was 400–4000 cm^−1^. In addition, adsorbent properties before and after dye adsorption were characterized by scanning electron microscopy (SEM) using a Zeiss FESEM Ultra Plus (ZEISS, Oberkochen, Germany).

### 2.4. Batch Adsorption Experiments

Adsorption experiments in batch mode were performed in an orbital shaking air bath (VWR-Cienytech, Santiago de Compostela, Spain) at a stirring rate of 210 rpm, 298.15 K, and natural pH (6.0, 5.9, and 5.8 for MB and 4.3, 4.0, and 3.5 for MG corresponding to 50, 250, and 500 mg·L^−1^, respectively). The experiments carried out consisted of the analysis of the initial dye concentration (between 50 and 500 mg L^−1^) and carbon dosage (between 0.2 and 1 g L^−1^) to study their effect on the removal of methylene blue and malachite green.

The amount of adsorbed dye in the supernatant after sample centrifugation was determined at different times at the maximum dye wavelength by UV/visible spectrophotometry (V630, Jasco, Hong Kong) and was used for calculating the dye removal efficiency (Equation (1)). Dye concentration in the aqueous phase was determined at 664 nm (for MB) and 616 nm (for MG). The adsorption capacity of the adsorbent (Equation (2)) was estimated as the amount of adsorbed dye per adsorbent mass used.

%adsorption = (C_0_ − C)/C_0_ · 100 (1)

q = (C_0_ − C) · V/m (2)

where C_0_ and C are the initial and final dye concentration, respectively (mg L^−1^), q is the adsorption capacity (mg g^−1^), V is the volume of the dye solution (L), and m is the adsorbent mass (g) employed in each experiment.

### 2.5. Adsorption Kinetics

Several models were employed to fit experimental data corresponding to the adsorption kinetics of both dyes under different experimental conditions. Thus, the pseudo-first-order, pseudo-second-order, and intraparticle diffusion models were evaluated to analyze the rate-limiting step of the overall process.

The pseudo-first-order kinetic model, recognized as the Lagergren equation, is as indicated in Equation (3).

log (q_e_ − q_t_) = log (q_e_)−(k_1_ · t)/2.303 (3)

where k_1_ (min^−1^) is the first-order reaction rate constant, q_t_ and q_e_ (mg·g^−1^) represent the adsorption capacity at time t and at the equilibrium.

The pseudo-second-order equation is expressed as Equation (4).

t/q_t_ = 1/(k_2_ · q_e_^2^) + t/q_e_ (4)

where k_2_ (g·mg^−1^·min^−1^) is the constant rate of the pseudo-second-order model.

Intraparticle diffusion equation (Weber–Morris model) is given by Equation (5).

q_t_ = k_id_ · √t + C (5)

where k_id_ is the intraparticle diffusion rate constant (mg·g^−1^·min^−0.5^), and C is the kinetic constant.

### 2.6. Adsorption Isotherms

The equilibrium data were obtained for both dyes from the adsorption experiments carried out at an adsorbent dosage of 0.5 g·L^−1^, natural pH, and 25 °C for 24 h. The specific amount of adsorbent previously defined was added to solutions with several initial dye concentrations in Erlenmeyer flasks. These flasks were stirred at 210 rpm and 25 °C in a shaking water bath (H20 SOW-Lauda, Burgwedel, Germany). The Langmuir (Equation (6)), Freundlich (Equation (7)), Temkin (Equation (8)), and Dubinin–Radushkevich (Equation (9)) models were used to fit equilibrium the experimental data.

q_e_ = (q_m,L_ · k_L_ · C_e_)/(1 + K_L_ · C_e_) (6)

q_e_ = k_F_ · Ce ^(1/n)^ (7)

q_e_ = B · ln (k_T_ · C_e_) (8)

q_e_ = q_m,D_ · exp (−k_DR_ · ε^2^) (9)

where C_e_ is the concentration of dye at equilibrium (mg·L^−1^); q_e_ is the adsorption capacity at equilibrium (mg·g^−1^); q_m_ is the maximum adsorption capacities (mg·g^−1^); K_L_ corresponds to the Langmuir adsorption constant (L·mg^−1^); K_F_ and n are Freundlich model constants; B is associated to the heat of adsorption (J·mol^−1^); K_T_ (L·g^−1^) is the other Temkin constants, R is the universal constant of gases (J·mol^−1^·K^−1^), T (K) is the absolute temperature; ε (kJ·mol^−1^) is the Polanyi coefficient; and K_DR_ is the activity coefficient (mol^2^·J^−2^).

### 2.7. Packed Bed Adsorption Simulation

Aspen Adsorption V14 was employed to carry out simulated breakthrough curves corresponding to MB and MG adsorption in almond shell activated carbon, varying different operation variables, such as wastewater flowrate, bed heigh, or dye concentration. Some assumptions were made for dye simulation based on the previous paper of Juela [13]. To complete this simulation procedure, some considerations from the results obtained in the previous sections were applied (i.e., equilibrium isotherm, mass transfer coefficient, etc.).

## 3. Results

### 3.1. Adsorbent Characterization

**Figure 1:**
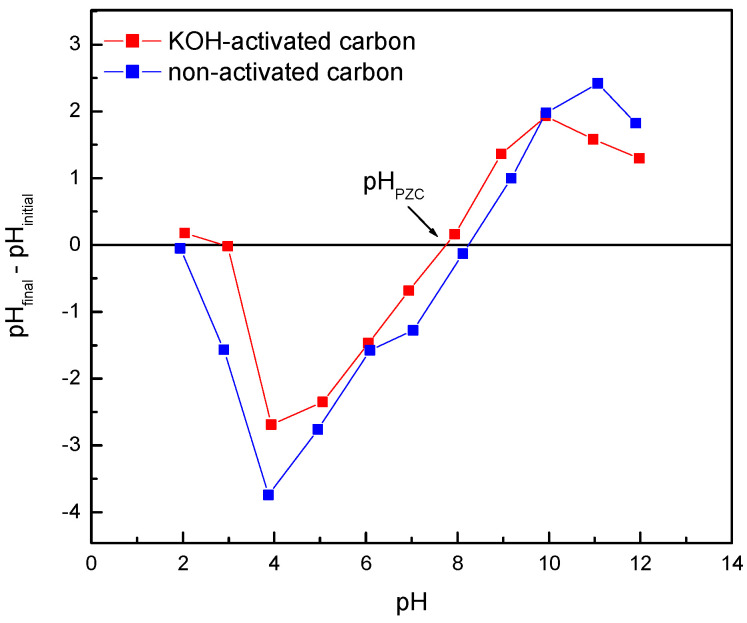
Point of zero charge (pH_pzc_) determination for almond shell carbons.

The value corresponding to the point of zero charge (pH_pzc_) can be determined by plotting ΔpH against the initial pH, as shown in Figure 1. The pH_pzc_ shows the point at which the electrostatic interactions (positive and negative) are equal. In this way, below this value (pH_pzc_), the surface of the adsorbent is positively charged, while above it will be negatively charged. The values obtained for carbons without and with activation were 8.3 and 7.8, respectively. Therefore, considering that the dyes used in this study were cationic, it would be expected that adsorption would be enhanced at a pH higher than pH_pzc_. However, in this first stage, it was decided to work at a natural pH to simplify the operation and reduce costs.

**Figure 2:**
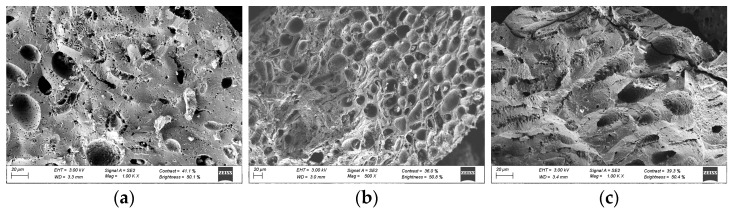
Activated carbon surface images obtained with scanning electron microscopy (SEM) before (**a**) and after methylene blue (**b**) and malachite green (**c**) adsorption (C_0_ = 250 mg·L^−1^, adsorbent dosage = 0.5 g·L^−1^, T = 25 °C, t = 1440 min, natural pH).

SEM images were used to analyze the texture and characteristics of the almond shell activated carbon surface. As an example, Figure 2 shows the images obtained by the SEM of the activated carbon before and after the adsorption of both dyes (MB and MG). The porous structure of the carbon (Figure 2a) is shown with pores of different sizes and shapes, probably due to activation with KOH [14]. Also, it seems that some of these pores are coated, and a layer is formed covering part of the surface (especially in Figure 2c) after adsorption, which confirms that both methylene blue and malachite green have been adsorbed on the carbon surface.

**Figure 3:**
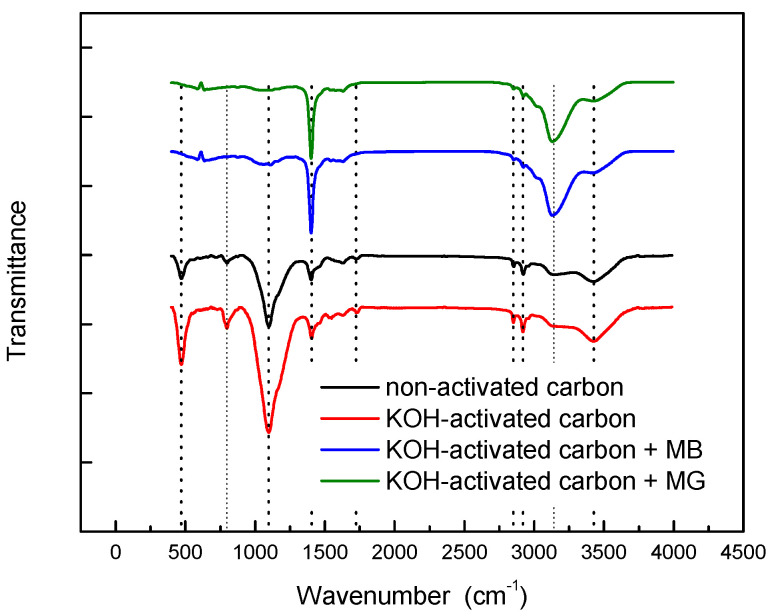
FTIR spectra of carbons before and after dye adsorption.

FTIR spectra for non-activated and activated carbon were recorded to evaluate their surface chemistry. As shown in Figure 3, both spectra were very similar, and the presence of different functional groups capable of participating in dyes adsorption was confirmed. The peaks at 470 and 797 cm^−1^ were associated with C–H groups, and the corresponding band at 1097 cm^−1^ could be assigned to the C-O stretching vibration of ether and alcohol groups, which indicates the presence of hydroxyl functional groups. At 1404 cm^−1^, a peak appeared, corresponding to C-N stretching. An additional band was observed at 1724 cm^−1^, confirming the presence of C = O groups. The peaks corresponding to C–H and N–H bonds were present at 2850 and 2919 cm^−1^, respectively. Finally, a wide band was observed at 3427 cm^−1^, which could be assigned to the hydroxyl and amine groups. As indicated, KOH activation did not cause significant changes in the functional groups on the surface, except for increasing the intensity of some peak and wavenumber shifts.

Figure 3 also shows the FTIR spectra of the activated carbon after the adsorption of both dyes used in the present study. Some changes were observed, mainly in the intensity of the bands. More specifically, changes were observed in the region 3100–3400 cm^−1^, with a decrease in the band at 3427 cm^−1^ and an increase in the corresponding one at 3140 cm^−1^, both related to hydroxyl and amine groups [15]. Also, an increase in the intensity of the signal at 1404 cm^−1^ (C-N stretching) was observed [16,17], as well as a decrease at 1097 cm^−1^ (C-O stretching).

In applications that involve the use of solid material, and especially when its porous structure could play an important role in the process, as is the case, the knowledge of some characteristics, such as specific surface area, is crucial and allows for a better understanding of the adsorption mechanism.

Therefore, the determination of the carbon-specific surface area was carried out using gas adsorption; specifically, the adsorption of nitrogen at 77 K and carbon dioxide at 273 K were performed. This determination is usually made with nitrogen, but in recent years, the complementary use of carbon dioxide has been included to provide more information on the pore structure in the range of smaller pores [18].

**Figure 4:**
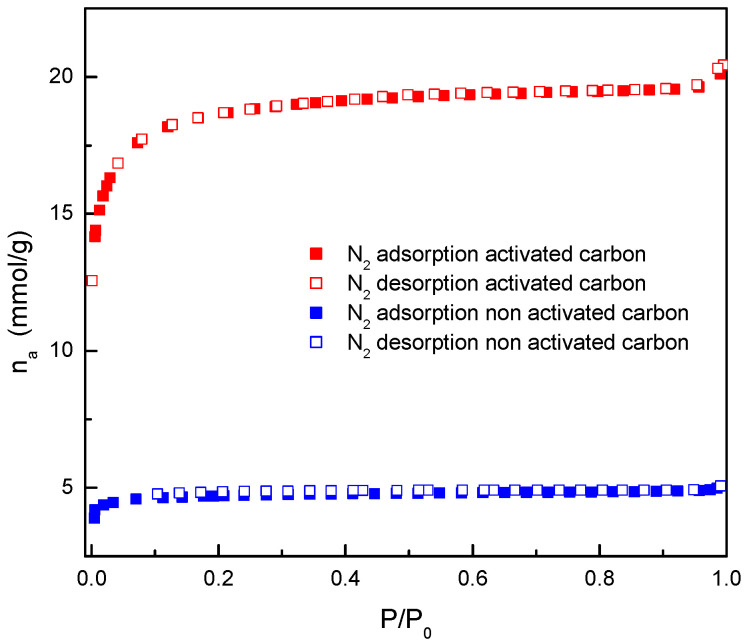
Nitrogen adsorption and desorption isotherms at 77 K for almond shell non-activated and activated carbons.

Figure 4 shows the experimental data of adsorption and desorption isotherms of nitrogen at 77 K, both for the activated carbon used for dye adsorption studies and for its carbonized precursor. It was observed that, as the relative pressure increases, and therefore, the presence of nitrogen molecules in the gas phase, there is an increase in the amount adsorbed on the materials, which is significantly higher for the activated carbon. This increase is important at low relative pressures, subsequently reaching a constant value.

Considering that the amount of adsorbed gas is directly related to the solid surface area, it can be concluded that the activated material has a greater surface area, which is the aim of the activation of carbonized materials.

A deeper analysis of the nitrogen adsorption/desorption isotherms (Figure 4) confirmed that both have a shape similar to the Langmuir isotherm and can be designated type I isotherms. This type of isotherm was observed in materials whose porous structure is formed mainly by small pores, included within the so-called micropores. The main difference observed was the range of relative pressure in which the knee was formed. In the case of the non-activated carbon, the knee formation occurred in a narrow range of relative pressures, which classifies this isotherm as type Ia. In this case, the pores had very small size, and therefore, the formation of several layers of nitrogen molecules inside was not favored. Regarding the activated carbon, the range of relative pressures was larger and was in accordance with the formation of micropores (due to the shape of isotherm) but in a larger size range. In this case, the isotherm corresponds to type Ib [19].

**Figure 5:**
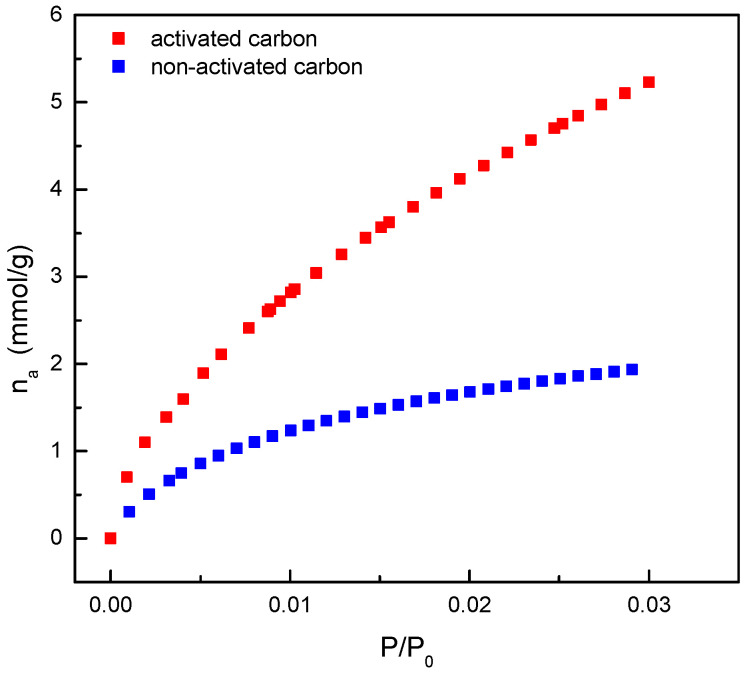
Carbon dioxide adsorption isotherms at 273 K for almond shell non-activated and activated carbons.

As mentioned previously, the study related to the characterization of the porous structure was completed, recording the carbon dioxide adsorption data at 273 K. These data are shown in Figure 5 for both non-activated and activated carbons.

A higher amount of carbon dioxide was adsorbed by the activated material as for nitrogen adsorption. Previous studies [18] have related the amount of adsorbed carbon dioxide to the specific surface area corresponding to ultra-microporosity (pores with diameters lower than 0.7 nm). As indicated, this type of study complements that using nitrogen at 77 K, since this one, due to the low temperature used, has mass transfer limitations in low-size pores due to increased viscosity. This fact causes a nitrogen size exclusion, and it does not allow for determination of the pore volume corresponding to diameters lower than 0.7 nm. Therefore, based on the results shown in Figure 5, the activated carbon also seems to have a higher surface area than the non-activated carbon in the ultra-micropore range.

The experimental data shown in Figure 4 and Figure 5 were used to calculate the specific surface area of the activated carbon for both nitrogen and carbon dioxide adsorption using the BET equation, which are shown in Table 1 together with other parameters related to its porous structure.

As shown, the values of the surface area determined using the BET equation lead to the conclusion that the activation process obtains a relatively high specific surface area, which can be considered as a suitable characteristic for the use of this material in dye adsorption.

In addition, pore size distribution in the porous structure of activated carbon can be considered another important characteristic for adsorption. The determination of pore size distribution was carried out with the NLDFT Advanced PSD tool included in the MicroActive software version 5.02 from Micromeritics. This tool allows us to obtain the pore size distribution by combining the nitrogen adsorption experiments at 77 K and carbon dioxide adsorption at 273 K.

**Figure 6:**
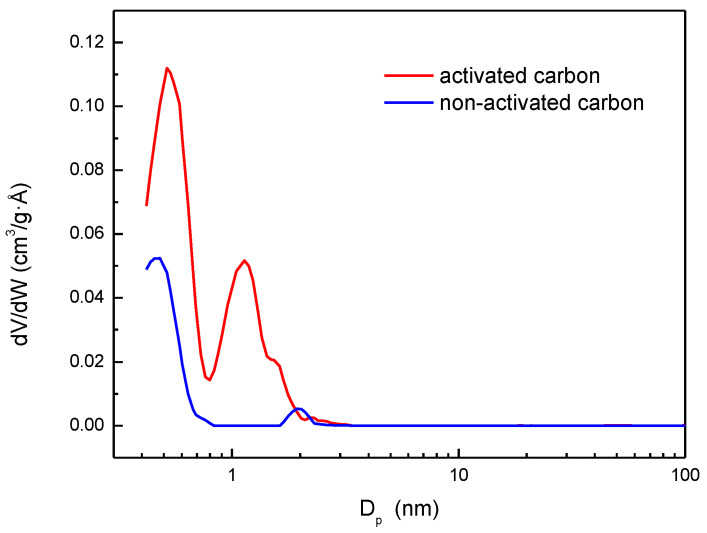
Pore size distribution of the almond shell activated carbon estimated using NLDFT model.

Figure 6 shows that most of the porous structure of the activated carbon is made up of micropores smaller than 2 nm and a small fraction of mesopores (sizes between 2 and 50 nm). These results agree with the conclusions previously obtained from the adsorption isotherm shape (Figure 4). Moreover, the large relative pressure range for the knee formation in the isotherm can be related with the second peak present in Figure 6 corresponding to supra-micropores (0.7–2 nm). The values of the other parameters shown in Table 1 also confirm that the material is highly microporous (78.2% microporosity). An important increase in the surface area and pore volume was observed when the activation was carried out.

### 3.2. Dye Adsorption Studies

#### 3.2.1. Effect of Adsorbent Dosage and Initial Dye Concentration

**Figure 7:**
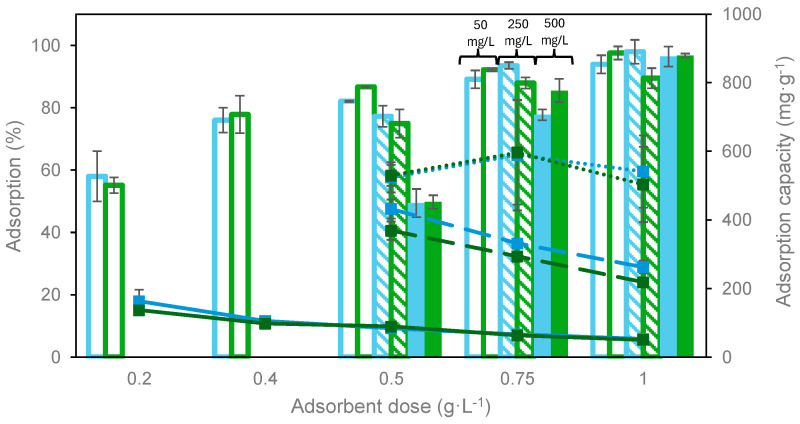
Influence of adsorbent dosage on adsorption percentage (columns) and capacity (symbols + lines) of MB (blue) and MG (green). T = 25 °C, t = 24 h. Solid line: 50 mg·L^−1^; dashed line: 250 mg·L^−1^; dotted line: 500 mg·L^−1^.

**Figure 8:**
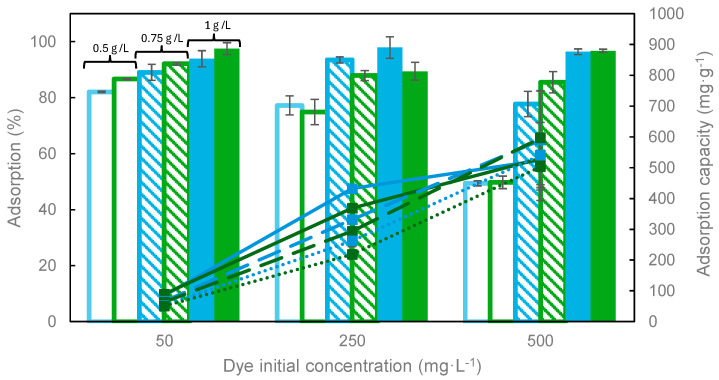
Influence of dye initial concentration on adsorption percentage (columns) and capacity (symbols + lines) of MB (blue) and MG (green). T = 25 °C, t = 24 h. Solid line: 0.5 g·L^−1^; dashed line: 0.75 g·L^−1^; dotted line: 1 g·L^−1^.

The influence of the adsorbent dosage on dye removal efficiency and adsorption capacity was analyzed at different initial dye concentrations. This information allows us to determine the best operating conditions to achieve the objectives established for dye recovery and evaluate the costs associated with the carbon. The experimental results obtained are shown in Figure 7 and Figure 8.

The results show that, for both dyes and at all initial concentrations tested (Figure 7), as the amount of adsorbent increases, there is a significant increase in the adsorption percentage reaching values above 95% at 1 g·L^−1^. This behavior is due to the greater number of active centers available due to the increased total surface area, which produces the shift of the equilibrium favoring the adsorption of a larger number of molecules on the activated carbon surface [17]. It can also be observed that for dye concentrations of 50 and 250 mg·L^−1^, small differences (lower than 5%) were found using 0.75 and 1 g·L^−1^ as the adsorbent dosages.

Regarding the influence of the adsorbent dosage on the adsorption capacity, in general, it can be concluded that, by increasing the carbon dosage, the adsorption capacity decreases for both dyes, except for the highest initial dye concentration. In this case, a maximum was observed at an adsorbent dosage of 0.75 g·L^−1^ that can be explained by the large amount of adsorbate molecules present in the liquid phase and more adsorption sites available in the carbon.

Figure 8 shows the influence of dye initial concentration on adsorption. In general, as expected, higher adsorption percentages were obtained at the lowest initial dye concentration. Moreover, for both dyes, a change in the trend was observed depending on the adsorbent dosage used; thus, when relatively low carbon dosages were used, the adsorption percentage was significantly higher using low dye concentrations, due to the lower presence of adsorbate molecules. Conversely, a significant increase in the adsorption percentage was observed when increasing the adsorbent dosage, reaching similar or even higher values at higher initial dye concentrations, probably related to the carbon/dye ratio. Furthermore, it can be seen that the carbon was slightly more effective for the removal of methylene blue than for malachite green.

#### 3.2.2. Adsorption Kinetics

**Figure 9:**
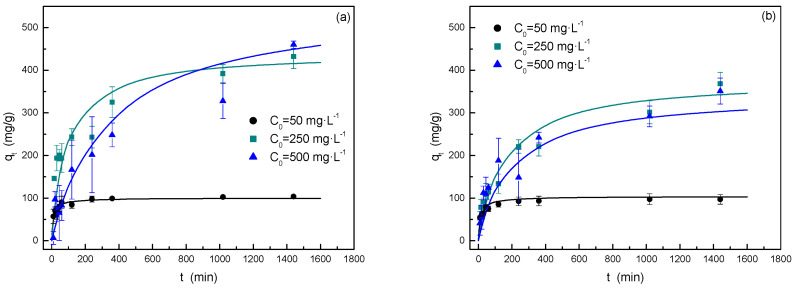
Adsorption kinetics for the adsorption of MB (**a**) and MG (**b**) adsorption by almond shell activated carbon using different initial dye concentrations at 25 °C, natural pH, and an adsorbent dosage of 0.5 g·L^−1^. Solid line corresponds to the fitting to the pseudo-second-order model.

As previously described, several experiments have been carried out at 25 °C and an adsorbent dosage of 0.5 g·L^−1^ to analyze the kinetic behavior of the dye adsorption process and to evaluate the main adsorption mechanism and determine the existence of limiting rate steps. In this way, different models have been tested to fit the experimental data as shown in Figure 9 and Table 2.

Figure 9 shows typical behavior in this type of adsorption system, in which, at short times, a high dye adsorption rate was observed; whereas, as the time increases, a decrease in the adsorption rate was produced due to an important increase in the adsorbent saturation degree [3]. Comparing both dyes, it was observed that MB adsorption is faster at short times, especially at an initial dye concentration of 250 mg·L^−1^. Additionally, for the highest initial dye concentration, the adsorption rate tends to decrease, mostly for MB, perhaps due to the repulsions between dye molecules that hinder the overall adsorption process. On the basis of this hypothesis and taking into account the larger size of MB in comparison to MG, the first dye can produce a difficulty in the diffusion in porous structure once part of molecules is adsorbed.

The adsorption kinetic data were fitted to the commonly used models, i.e., the pseudo-first-order (PFO), the pseudo-second-order (PSO), and the intraparticle diffusion models (IP). At first, the use of the PFO model was discarded, because the fitting parameters were unsatisfactory. This model is considered suitable for modelling adsorption processes in which the limiting stage is the mass transfer of adsorbate from the bulk to the external surface of the adsorbent material [17]. Therefore, this conclusion could agree with the fact that the main part of the surface area in the carbon is due to the porous structure, and the diffusion processes inside the pores can be more important than the external diffusion. For this reason, the other models employed have been analyzed deeply.

Table 2 includes the fitting parameters of both models. In general, the PSO model has shown a better fit for both initial dye concentrations than the IP one. These results lead us to conclude the important role of the dyes’ adsorption step over the carbon surface (chemisorption process) that is the rate-limiting step. In relation to the fitting parameters included in Table 2, k_2_ values for MB adsorption are generally higher than the corresponding ones for MG adsorption, which agrees with the previous analysis.

Otherwise, the IP model has confirmed that, except for MG at 50 mg·L^−1^, two steps are involved in the dye adsorption mechanism, i.e., the mass transfer of dyes from solution to external carbon surface and the internal diffusion in the porous structure, as multilinearity was observed by the plots q_t_ vs. √t. Considering the values of k_id_, the diffusion in the bulk phase was the fastest stage, and therefore, the diffusion into the pores was probably the rate-limiting stage in the mesopores and micropores and especially for MB for its higher molecular size, as mentioned previously.

#### 3.2.3. Adsorption Equilibrium

**Figure 10:**
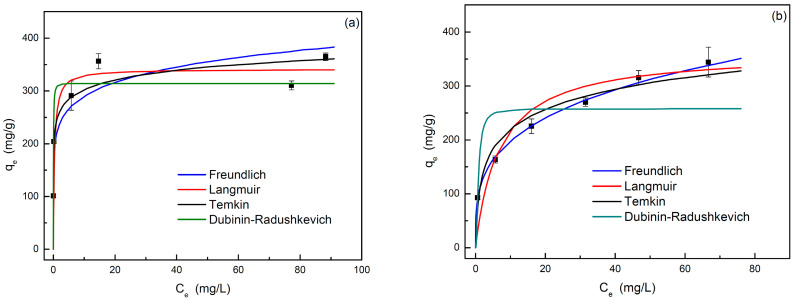
Experimental data and adsorption isotherm models corresponding to (**a**) MB and (**b**) MG adsorption by almond shell activated carbon at 25 °C, natural pH, and adsorbent dosage of 0.5 g·L^−1^.

The analysis of adsorption isotherms is very useful information for allowing us to evaluate separation processes based on adsorption. Figure 10 shows the experimental equilibrium data obtained for each dye at selected conditions. At first glance, some differences can be observed in the shape of the isotherms obtained, since, in the case of MB, the adsorbed amount tends to be a constant value when the liquid phase concentration reaches 20 mg·L^−1^. However, in the case of MG, this plateau is not reached; thus, a monotonic increase in the amount of adsorbed dye was observed, and then, the saturation of the adsorbent is not reached.

Based on the previously described behavior, it seems that the carbon has a strong affinity for MB. Once the saturation of the carbon was reached, an increase in the concentration of MB in the fluid phase was observed. This behavior corresponds to a type H2 isotherm according to the Giles classification. In the case of MG, the affinity towards the carbon is significantly lower, and the isotherm would be classified as type L2, in which the interactions between the dye molecules could be important, increasing the resistance to adsorption [20]. The experimental equilibrium data were fitted with different models previously described (Figure 10). Table 3 shows the fit parameters, as well as the goodness of the fit to each model. In view of the results, it can be concluded that the Langmuir and Freundlich models showed the best fit for MB and MG equilibrium data, respectively, which is in agreement with the previous discussion about the different shape of the adsorption isotherms. The Temkin model also fits the experimental data for both dyes with suitable results.

In any case, the Langmuir isotherm can satisfactorily fit the adsorption equilibrium for both dyes, and it allows us to conclude that the main part of adsorption occurs through the formation of a monolayer. Also, for both dyes, the parameter n of the Freundlich model reaches values between 1 and 10, which indicates a favorable adsorption process [21]. The higher value in the case of MB adsorption agrees with the higher affinity of the carbon for this dye previously commented. Temkin parameters also allow us to characterize the adsorption phenomenon. Temkin adsorption potential (B_T_·lnK_T_) reached values of 0.42 and 0.47 kJ·mol^−1^ for MB and MG, respectively, that are lower than 8 kJ·mol^−1^ associated to physical adsorption. In relation with the value of the b_T_ parameter (b_T_ = R·T/B_T_) for MB (93.8 kJ·mol^−1^) and MG (46.5 kJ·mol^−1^), MB adsorption reached values higher than 80 kJ·mol^−1^, concluding the existence of chemical adsorption. This contradiction between both parameters indicates that the Temkin model is not conclusive [22]. On the other hand, the free energy of sorption calculated using Dubinin–Radushkevich fitting parameters were 7.7 and 1.7 kJ·mol^−1^ for MB and MG, respectively. These values do not reach 8 kJ·mol^−1^, indicating a higher influence of physical processes in the overall adsorption phenomenon. However, data corresponding to MB adsorption are close to the energy range associated to chemical adsorption in agreement with the better behavior of the Langmuir isotherm for modelling MB adsorption.

Table 4 shows a comparison of the adsorption capacity obtained using the Langmuir model for both dyes in the present work with those of previous studies using different biomass-derived activated carbons. It can be seen that the almond shell activated carbon exhibited high adsorption capacities for both dyes but particularly for MG, which far surpassed all the other materials.

**Figure 11:**
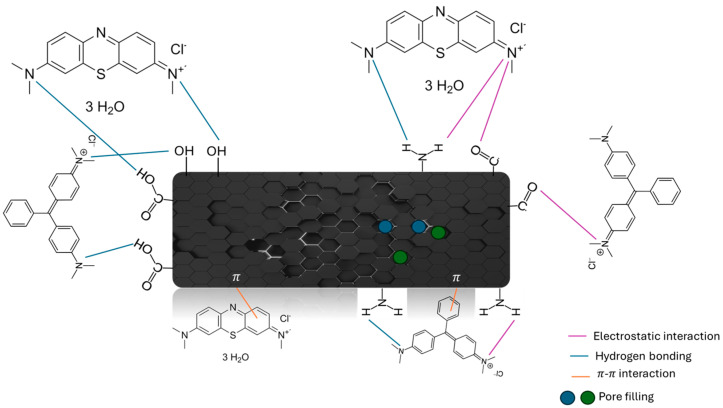
Proposal of the interactions involved in the adsorption mechanism for MB and MG adsorption.

Based on the previously discussed experimental data, Figure 11 shows a proposal of the adsorption mechanism of MB and MG over this type of biomass-based carbon. FTIR spectra previously analyzed in Figure 3 agree with the proposed mechanism enhancing the interactions between nitrogen present in carbon and dyes with different functional groups: decreasing the band at 3400 cm^−1^ (N-H) and enhancing the band at 1402 cm^−1^ (N-O).

### 3.3. Simulation of Dyes Adsorption in a Packed-Bed

The different studies developed in this work focused on the adsorption of two dyes (MB and MG) on an activated carbon produced from a biomass waste almond shell and have been completed with a simulation of the separation of these pollutants in a packed-bed column. For this, the Aspen Adsorption V14 software package from Aspentech has been used. Moreover, with the objective of validating the methodology and carrying out a comparison of MB adsorption dynamics, a previous study has been used [34]. Based on previous studies about steady-state simulation for the adsorption of different pollutants (including dyes), the NRTL model has been chosen [35]. Furthermore, Table 5 shows the most important parameters used in the present study. The values of the mass transfer coefficients for both dyes have been estimated based on the adsorption kinetics previously analyzed in Section 3.2.2. The isotherm models for each dye employed in the software have been those that showed the best fit in Section 3.2.3, that is, the Langmuir model for MB and the Freundlich model for MG.

**Figure 12:**
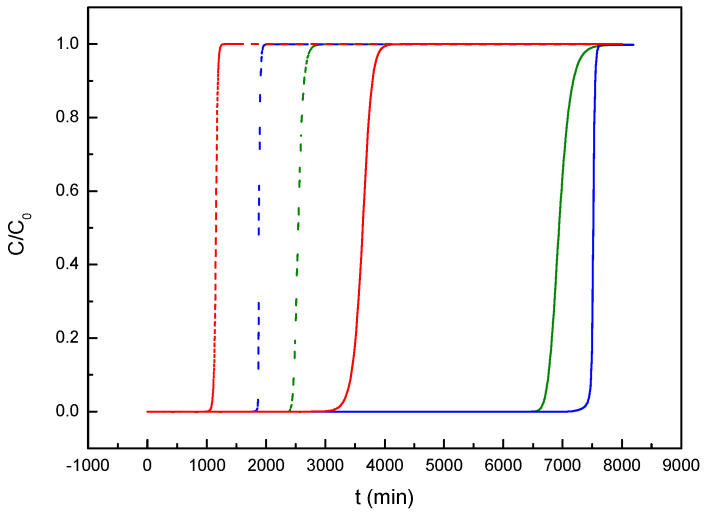
Simulated breakthrough curves for dyes adsorption by activated carbon: Red curves, MB adsorption in coconut shell carbon [34]. Blue curves, MB adsorption in almond shell carbon. Green curves, MG adsorption in almond shell carbon. Continuous lines: 50 mg·L^−1^, initial dye concentration. Dashed line: 200 mg·L^−1^, initial dye concentration. Flowrate, Q_L_ = 0.01 mL·min^−1^. Mass of carbon, m_b_ = 12.1 g.

Once the data were collected or estimated, the simulation of the breakthrough curves for MB adsorption by almond shell activated carbon was carried out at two different initial concentrations and compared to those obtained for a coconut shell activated carbon [34], observing a very good agreement (Figure 12). Regarding the influence of the initial concentration, when it was increased, the breakthrough point was reached in a shorter time due to the high amount of dye molecules that were being fed to the adsorbent bed. On the other hand, it can be concluded that the almond shell carbon is able to adsorb a larger amount of MB than the coconut shell one, which can be related with its significantly largest surface area (1577 m^2^·g^−1^ vs. 1026 m^2^·g^−1^), allowing for an increase in the adsorption capacity.

Various simulations were also carried out to analyze the role that different operating variables may have on the shape of the breakthrough curves, since they can provide valuable information for the industrial application of this type of operation. Hence, Figures S1 and S2 show the influence of the adsorbent bed height and initial dye concentration on the breakthrough curves of MB adsorption in a fixed bed.

When the bed length was increased, the breakthrough curve shifted to longer times, because there was a greater amount of carbon surface available for dye adsorption. The opposite behavior was observed when the influent flowrate was increased as the adsorbent saturation occurred in a shorter time due to the increase in the amount of MB molecules fed per unit of time. In both studies, no notable changes were observed in the shape of the breakthrough curve, with a similar mass transfer zone (MTZ) in all cases.

The same study was carried out for green malachite adsorption (MG), obtaining a similar behavior to that previously explained for MB (Figures S3–S5).

Figure 12 also shows a comparison of the breakthrough curves obtained for both dyes (MB and MG) using different inlet concentrations. It was observed that the shape of the curve for MG changed when the breakthrough time increased (by decreasing the concentration of dye in the feed), showing a clear increase in MTZ. Conversely, in the case of MB, the shape of the curve remained relatively constant.

Moreover, it can be observed that for the lower dye inlet concentration, dye adsorption on the carbon was higher for MB; whereas, the opposite occurred when the dye inlet concentration was increased. This behavior can be related to the adsorption isotherms previously obtained in relation to the affinity of the dyes for the carbon and the type of interactions that predominate for each system. In addition, the fact that MTZ increased notably for MG (especially for low dye concentrations) could be related to interactions between the dye molecules that hinder their adsorption on the carbon surface, and therefore, the length of the MTZ tends to increase.

## 4. Conclusions

The present study analyzed the use of one activated carbon derived from an industrial waste (almond shell) for the adsorption of two dyes, blue methylene and green malachite, from aqueous solutions. Activation with KOH led to an important increase in both surface area and pore size, which favors the adsorption of this type of pollutants. Regarding the influence of adsorption conditions, it was found that, by increasing the adsorbent dosage, the adsorption percentage (for all tested dye initial concentrations) increased but caused a decrease in the adsorption capacity, due to limitations caused by the adsorption equilibrium. The behavior is slightly different for the highest concentration of dyes used (500 mg·L^−1^), because a maximum was reached at a dosage of 0.75 g·L^−1^. On the other hand, a greater affinity of the carbon for MB than for MG was detected.

Adsorption kinetics was explained by the pseudo-second-order kinetic model (PSO), suggesting chemical adsorption as the main adsorption mechanism; although, the importance of intraparticle diffusion in the overall adsorption mechanism was also evidenced. Regarding the adsorption equilibrium, the Langmuir and Freundlich models showed the best performance, particularly Langmuir for MB and Freundlich for MG. This difference in the adsorption equilibrium of both dyes can be related to the interactions between dye molecules, which are more significant for MG. The adsorbent fabricated in the present work reached high values of adsorption capacity compared to carbons from other biomass sources.

Finally, simulation studies (including the previously obtained data for equilibrium and kinetic studies) have shown that the activated carbon prepared in the present study presented a good overall performance for both dyes’ adsorption. Furthermore, the most pronounced interactions between MG molecules explain the different shape of the breakthrough curves compared to the MB ones, increasing the MTZ and decreasing the adsorption at low dye concentrations in the fluid phase.

## Figures and Tables

**Table 1:** Porous structure characteristics of almond shell carbons.

Parameter	Non-Activated Carbon	Activated Carbon
Surface area (m^2^/g) N_2_ at 77 K (BET)	294 ± 7	1577 ± 13
Average pore size (Å) N_2_ at 77 K (BET)	21.8	17.7
Surface area (m^2^/g) CO_2_ at 273 K (BET)	268 ± 1	772 ± 12
Total pore volume (cm^3^/g) at P/P_o_ = 0.99	0.161	0.697
Microporosity (%)	92.7	78.2

**Table 2:** Fit parameters of kinetic models for MB and MG adsorption by almond shell activated carbon at 25 °C, natural pH, and an adsorbent dosage of 0.5 g·L^−1^.

Kinetic Model	Parameter	Methylene Blue	Malachite Green
C_0_ = 50 mg·L^−1^			
Adsorption half-time	t_1/2_ (min)	10.9	13.8
Pseudo-second-order	q_e_ (mg·g^−1^)	100.4	104.0
	k_2_ (g·mg^−1^·min^−1^)	9.16·10^−4^	6.96·10^−4^
	R^2^	0.9998	0.9998
Intraparticle diffusion	k_id,1_ (mg·g^−1^·min^0.5^)	11.87	14.68
	C_1_ (mg·g^−1^)	7.55	1.77
	R^2^	0.9243	0.9753
	k_id,2_ (mg·g^−1^·min^0.5^)	0.286	3.02
	C_2_ (mg·g^−1^)	93.03	53.14
	R^2^	0.6673	0.9075
	k_id,3_ (mg·g^−1^·min^0.5^)	-	0.273
	C_3_ (mg·g^−1^)	-	93.41
	R^2^	-	0.8709
C_0_ = 250 mg·L^−1^			
Adsorption half-time	t_1/2_ (min)	86.5	150.1
Pseudo-second-order	q_e_ (mg·g^−1^)	441.1	378.6
	k_2_ (g·mg^−1^·min^−1^)	2.62·10^−5^	1.76·10^−5^
	R^2^	0.9880	0.9747
Intraparticle diffusion	k_id,1_ (mg·g^−1^·min^0.5^)	35.85	13.02
	C_1_ (mg·g^−1^)	1.69	13.12
	R^2^	0.9974	0.9619
	k_id,2_ (mg·g^−1^·min^0.5^)	7.65	7.55
	C_2_ (mg·g^−1^)	148.3	73.23
	R^2^	0.9640	0.9769
	k_id,3_ (mg·g^−1^·min^0.5^)	-	-
	C_3_ (mg·g^−1^)	-	-
	R^2^	-	-
C_0_ = 500 mg·L^−1^			
Adsorption half-time	t_1/2_ (min)	327.1	189.2
Pseudo-second-order	q_e_ (mg·g^−1^)	551.9	343.2
	k_2_ (g·mg^−1^·min^−1^)	5.54·10^−6^	1.54·10^−5^
	R^2^	0.9966	0.9933
Intraparticle diffusion	k_id,1_ (mg·g^−1^·min^0.5^)	10.43	11.46
	C_1_ (mg·g^−1^)	22.47	20.48
	R^2^	0.9397	0.8573
	k_id,2_ (mg·g^−1^·min^0.5^)	1.25	3.31
	C_2_ (mg·g^−1^)	419.7	205.9
	R^2^	0.7235	0.9763
	k_id,3_ (mg·g^−1^·min^0.5^)	-	-
	C_3_ (mg·g^−1^)	-	-
	R^2^	-	-

**Table 3:** Equilibrium parameters of different models for the adsorption of methylene blue and malachite green.

Equilibrium Model	Parameter	Methylene Blue	Malachite Green
Langmuir	q_m,L_ (mg·g^−1^)	341.3	363.6
	k_L_ (L·mg^−1^)	2.64	0.15
	R^2^	0.989	0.982
Freundlich	N	7.9	3.6
	k_F_ (mg·g^−1^·(L·mg^−1^)^−1/n^)	217.0	103.5
	R^2^	0.896	0.998
Temkin	B_T_ (J·mol^−1^)	26.4	53.2
	k_T_ (L·mg^−1^)	9514.8	6.3
	R^2^	0.910	0.948
Dubinin–Radushkevich	q_m,DR_ (mg·g^−1^)	314.2	257.6
	k_DR_ (mol^2^·kJ^−2^)	8.54·10^−9^	1.83·10^−7^
	R^2^	0.919	0.730

**Table 4:** Langmuir adsorption capacity of several biomass-derived activated carbons for MB and MG at 25 °C.

Precursor Material	Dye	Capacity (mg·g^−1^)	S/L (g·L^−1^)	Reference
Almond shell	MB	341	0.5	This work
Walnut shell	MB	247	0.5	[4]
Rubber seed pericarp	MB	348	0.6	[23]
Oil palm frond and palm kernel shell	MB	332	2.5	[24]
Sunflower pith	MB	581	1.0	[25]
Chickpea peel	MB	200	0.8	[26]
Sugarcane bagasse waste	MB	142	5.0	[27]
Almond shell	MG	364	0.5	This work
Charcoal	MG	27	0.4	[28]
Pinus roxburghii cone	MG	250	0.7	[29]
Walnut shell	MG	155	0.6	[30]
Okra stalks	MG	100	1.0	[31]
Hevea brasiliensis root	MG	260	1.0	[32]
Peach pit	MG	70	2.0	[33]

**Table 5:** Modelling parameters for dye adsorption simulation.

Parameter	Value
Internal diameter of packed bed (cm)	2.4
Interparticle voidage (m^3^ void·m^−3^ bed) [36]	0.497
Solid density (kg·m^−3^)	3045 [36]
Mass transfer coefficient MB (s^−1^)	0.00103
Mass transfer coefficient MG (s^−1^)	0.00126
Mass of carbon (g)	12.1

## Data Availability

The original contributions presented in this study are included in the article/Supplementary Materials. Further inquiries can be directed to the corresponding author.

## Supplementary Materials

The following supporting information can be downloaded at: https://www.mdpi.com/article/10.3390/ma17246077/s1, Figure S1. Influence of bed length on the breakthrough curves of MB adsorption. Q_L_ = 0.01 mL·min^−1^, C_0_ = 200 mg·L^−1^, m_b_ = 12.1 g, and D_b_ = 2.4 cm. Figure S2. Influence of feed flowrate on the breakthrough curves of MB adsorption. C_0_ = 200 mg·L^−1^, m_b_ = 12.1 g, D_b_ = 2.4 cm, and H_b_ = 1.74 cm. Figure S3. Influence of dye concentrations in the feed stream on the breakthrough curves of MG adsorption. Q_L_ = 0.01 mL·min^−1^, m_b_ = 12.1 g, and D_b_ = 2.4 cm. Figure S4. Influence of bed length on the breakthrough curves of MG adsorption. Q_L_ = 0.01 mL·min^−1^, C_0_ = 200 mg·L^−1^, m_b_ = 12.1 g, and D_b_ = 2.4 cm. Figure S5. Influence of liquid phase flowrate on the breakthrough curves of MG adsorption. C_0_ = 200 mg·L^−1^, m_b_ = 12.1 g, D_b_ = 2.4 cm, and H_b_ = 1.74 cm.

## References

[1] Lotfy H.R., Roubik H. (2023). Water purification using activated carbon prepared from agriculture waste—Overview of a recent development. Biomass Convers. Biorefin..

[2] Ullah Khan W., Ahmed S., Dhoble Y., Madhav S. (2023). A critical review of hazardous waste generation from textile industries and associated ecological impacts. J. Indian Chem. Soc..

[3] Shakoor S., Nasar A. (2017). Adsorptive treatment of hazardous Methylene Blue dye from artificially contaminated water using Cucumis sativus peel waste as a low-cost adsorbent. Groundw. Sustain. Dev..

[4] Wisniewska M., Urban T., Tokarska K., Marciniak P., Giel A., Nowick P. (2024). Removal of organic dyes, polymers and surfactants using carbonaceous materials derived from walnut shells. Materials.

[5] Santos D.H.S., Duarte J.L.S., Tonholo J., Meili L., Zanta C.L.P.S. (2020). Saturated activated carbon regeneration by UV-light, H_2_O_2_ and Fenton reaction. Sep. Purif. Technol..

[6] Santos D.H.S., Xiao Y., Chaukura N., Hill J.M., Selvasembian R., Zanta C.L.P.S., Meili L. (2022). Regeneration of dye-saturated activated carbon through advanced oxidative processes: A review. Heliyon.

[7] Plassard F., Winiarski T., Petit-Ramel M. (2000). Retention and distribution of three heavy metals in a carbonated soil: Comparison between batch and unsaturated column studies. J. Contam. Hydrol..

[8] Kumar D., Pandey L.K., Gaur J. (2016). Metal sorption by algal biomass: From batch to continuous system. Algal Res..

[9] Selambakkannu S., Othman N.A.F., Bakar K.A., Karim Z.A. (2019). Adsorption studies of packed bed column for the removal of dyes using amine functionalized radiation induced grafted fiber. SN Appl. Sci..

[10] Li L., Sun F., Gao J., Wang L., Pi S., Zhao G. (2018). Broadening the pore size of coal-based activated carbon via a washing-free chem-physical activation method for high-capacity dye adsorption. RSC Adv..

[11] Amran F., Zaini M.A.A. (2020). Effects of chemical activating agents on physical properties of activated carbons—A commentary. Water Prac. Technol..

[12] Carabineiro S.A.C., Thavorn-Amornsri T., Pereira M.F.R., Serp P., Figueiredo J.L. (2012). Comparison between activated carbon, carbon xerogel and carbon nanotubes for the adsorption of the antibiotic ciprofloxacin. Catal. Today.

[13] Juela D.M. (2020). Comparison of the adsorption capacity of acetaminophen on sugarcane bagasse and corn cob by dynamic simulation. Sustain. Environ. Res..

[14] Pimentel C.H., Díaz-Fernández L., Gómez-Díaz D., Freire M.S., González-Álvarez J. (2023). Separation of CO_2_ using biochar and KOH and ZnCl_2_ activated carbons derived from pine sawdust. J. Environ. Chem. Eng..

[15] Sayğili H., Güzel F. (2018). Usability of activated carbon with well-developed mesoporous structure for the decontamination of malachite green from aquatic environments: Kinetic, equilibrium and regeneration studies. J. Porous Mater..

[16] Thang N.H., Khang D.S., Hai T.D., Nga D.T., Tuan P.D. (2021). Methylene blue adsorption mechanism of activated carbon synthesised from cashew nut shells. RSC Adv..

[17] Freire M.S., Gómez Díaz D., González Álvarez J., Pimentel C.H. (2023). Preparation of activated carbon from pine (*Pinus radiata*) sawdust by chemical activation with zinc chloride for wood dye adsorption. Biomass Convers. Biorefin..

[18] Thommes M., Kaneko K., Neimark A.V., Olivier J.P., Rodríguez-Reinoso F., Rouquerol J., Sing K.S.W. (2015). Physisorption of gases, with special reference to the evaluation of surface area and pore size distribution. Pure Appl. Chem..

[19] Manyá J.J., González B., Azuara M., Arner G. (2018). Ultra-microporous adsorbents prepared from vine shoots-derived biochar with high CO_2_ uptake and CO_2_/N_2_ selectivity. Chem. Eng. J..

[20] Giles C.H., MacEwan T., Nakhwa S., Smith D. (1960). A system of classification of solution adsorption isotherms and its use in diagnosis of adsorption mechanisms and in measurement of specific surface area solids. J. Chem. Soc..

[21] Spessato L., Bedin K.C., Cazetta A.L., Souza I.P.A.F., Duarte V.A., Crespo L.H.S., Silva M.C., Pontes R.M., Almeida V.C. (2019). KOH-super activated carbon from biomass waste: Insights into the paracetamol adsorption mechanism and thermal regeneration cycles. J. Hazard. Mater..

[22] Shikuku V.O., Mishra T. (2021). Adsorption isotherm modeling for methylene blue removal onto magnetic kaolinite clay: A comparison of two-parameter isotherms. Appl. Water Sci..

[23] Jawad A.H., Malek N.N.A., Khadiran T., Alothman Z.A., Yaseen Z.M. (2022). Mesoporous high-surface-area activated carbon from biomass waste via microwave-assisted-H_3_PO_4_ activation for methylene blue dye adsorption: An optimized process. Diam. Relat. Mat..

[24] Jasri K., Abdulhameed A.S., Jawad A.H., Alothman Z.A., Yousef T.A., Al Duaij O.K. (2023). Mesoporous activated carbon produced from mixed wastes of oil palm frond and palm kernel shell using microwave radiation-assisted K_2_CO_3_ activation for methylene blue dye removal: Optimization by response surface methodology. Diam. Relat. Mat..

[25] Baysal M., Bilge K., Yılmaz B., Papila M., Yürüm Y. (2018). Preparation of high surface area activated carbon from waste-biomass of sunflower piths: Kinetics and equilibrium studies on the dye removal. J. Environ. Chem. Eng..

[26] Jahan K., Singh V., Mehrotra N., Rathore K., Verma V. (2023). Development of activated carbon from KOH activation of pre-carbonized chickpea peel residue and its performance for removal of synthetic dye from drinking water. Biomass Convers. Biorefin..

[27] Jawad A.H., Abdulhameed A.S., Bahrudin N.N., Hum N.N.M.F., Surip S.N., Syed-Hassan S.S.A., Yousif E., Sabar S. (2021). Microporous activated carbon developed from KOH activated biomass waste: Surface mechanistic study of methylene blue dye adsorption. Water Sci. Technol..

[28] Ali F., Bibi S., Ali N., Ali Z., Said A., Wahab Z.U., Bilal M., Iqbal H.M.N. (2020). Sorptive removal of malachite green dye by activated charcoal: Process optimization, kinetic, and thermodynamic evaluation. Case Stud. Chem. Environ. Eng..

[29] Sharma G., Sharma S., Kumar A., Naushad M., Du B., Ahamad T., Ghfar A.A., Alqadami A.A., Stadler F.J. (2019). Honeycomb structured activated carbon synthesized from Pinus roxburghii cone as effective bioadsorbent for toxic malachite green dye. J. Water Process Eng..

[30] Merrad S., Abbas M., Trari M. (2023). Adsorption of malachite green onto walnut shells: Kinetics, thermodynamic, and regeneration of the adsorbent by chemical process. Fibers Polym..

[31] Yildiz H., Gülşen H., Şahin Ö., Baytar O., Kutluay S. (2023). Novel adsorbent for malachite green from okra stalks waste: Synthesis, kinetics and equilibrium studies. Int. J. Phytoremediat..

[32] Ahmad A.A., Ahmad M.A., Yahaya N.K.E.M., Karim J. (2021). Adsorption of malachite green by activated carbon derived from gasified Hevea brasiliensis root. Arab. J. Chem..

[33] Qu W., Yuan T., Yin G., Xu S., Zhang Q., Su H. (2019). Effect of properties of activated carbon on malachite green adsorption. Fuel.

[34] Zainol N.B. (2021). Aspen Simulation of Methylene Blue Adsorption Using Coconut Shell Based Activated Carbon. Bachelor’s Thesis.

[35] Hameed A., Hameed B.H., Almomani F.A., Usman M., Ba-Abbad M.M., Khraisheh M. (2024). Dynamic simulation of lead (II) metal adsorption from water on activated carbons in a packed-bed column. Biomass Convers. Biorefin..

[36] Rahimi V., Ferreiro-Salgado A., Gómez-Díaz D., Freire M.S., González-Álvarez J. (2024). Evaluating the performance of carbon-based adsorbents fabricated from renewable biomass precursors for post-combustion CO_2_ capture. Sep. Purif. Technol..

