# Supplementary material for: Evaluation of Almond Shell Activated Carbon for Dye (Methylene Blue and Malachite Green) Removal by Experimental and Simulation Studies
Source: Materials (Basel). 2024 Dec 12;17(24):6077. doi: 10.3390/ma17246077 (PMC11728400; doi:10.3390/ma17246077)
Supplement: Supplementary file 1 [file materials-17-06077-s001.zip › materials-3318599-supplementary.pdf]

Evaluation of Almond Shell Activated Carbon for Dyes (Methylene Blue and Malachite Green) Removal by Experimental and Simulation Studies

Adrián Rial [1], Catarina Helena Pimentel [1,2], Diego Gómez-Díaz [1] *, María Sonia Freire [1], Julia González-Álvarez [1]

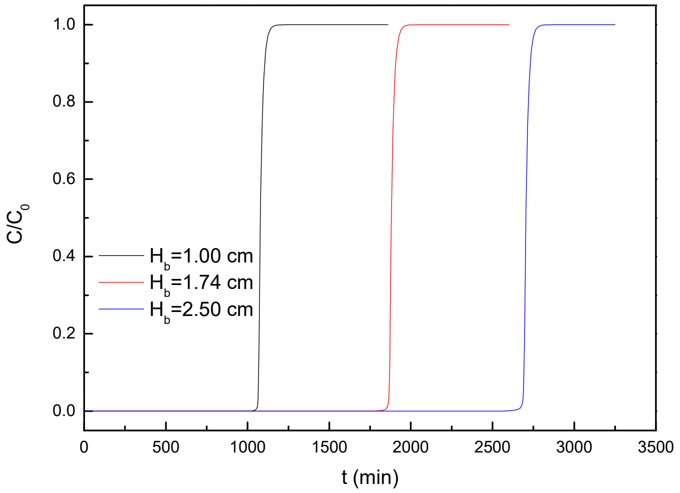

Figure S1. Influence of bed length upon the breakthrough curves of MB adsorption. Q_L = 0.01 mL·min^{-1}, C_0 = 200 mg·L^{-1}, m_b = 12.1 g, D_b = 2.4 cm.

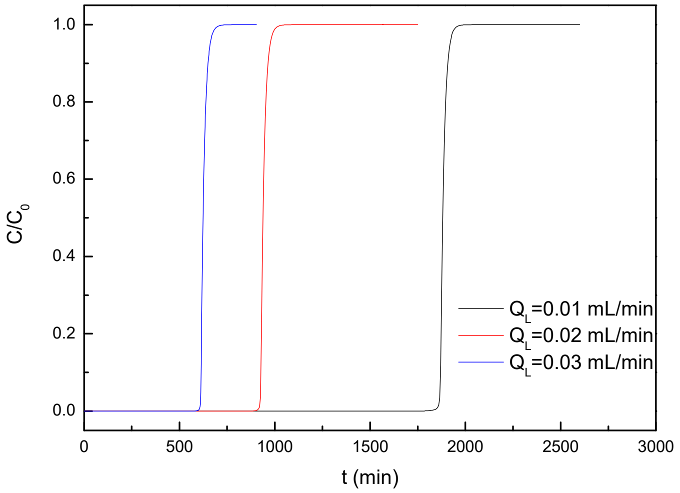

Figure S2. Influence of feed flowrate upon the breakthrough curves of MB adsorption. C_0 = 200 mg·L^{-1}, m_b = 12.1 g, D_b = 2.4 cm. H_b = 1.74 cm

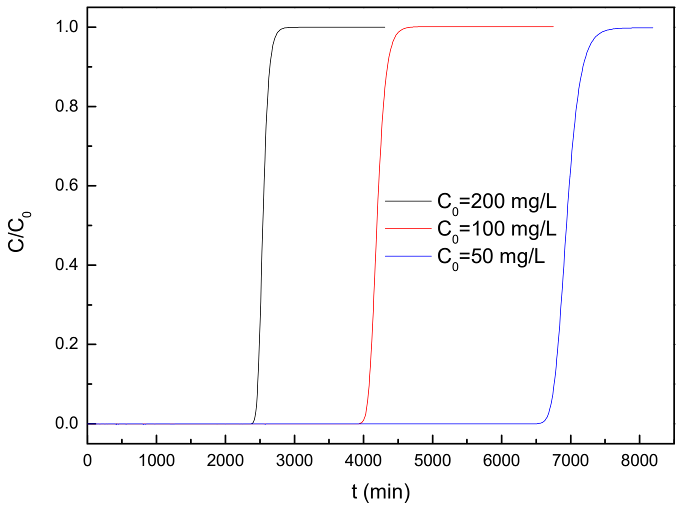

Figure S3. Influence of dye concentration in feed stream upon the breakthrough curves of MG adsorption. Q_L = 0.01 mL·min^{-1}, m_b = 12.1 g, D_b = 2.4 cm.

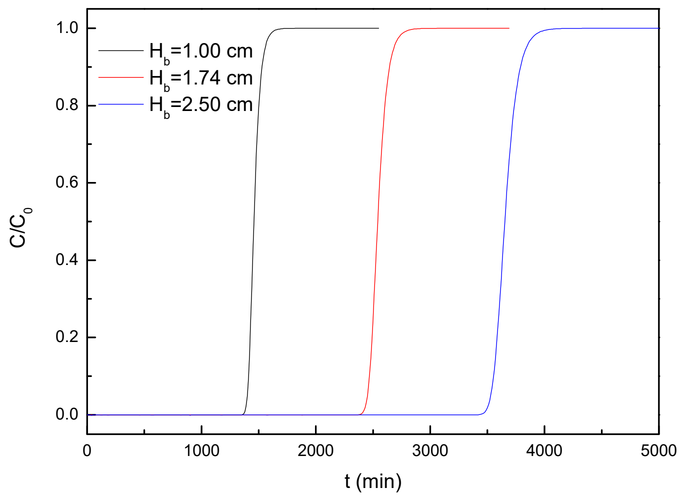

Figure S4. Influence of bed length upon the breakthrough curves of MG adsorption. Q_L = 0.01 mL·min^{-1}, C_0 = 200 mg·L^{-1}, m_b = 12.1 g, D_b = 2.4 cm.

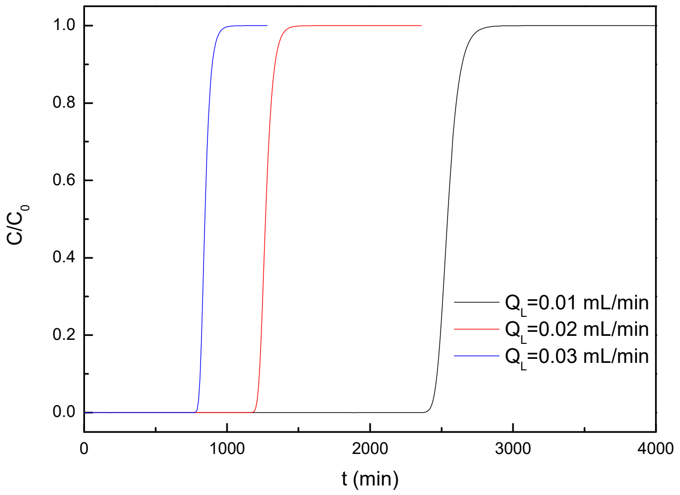

Figure S5. Influence of liquid phase flowrate upon the breakthrough curves of MG adsorption. C_0 = 200 mg·L^{-1}, m_b = 12.1 g, D_b = 2.4 cm. H_b = 1.74 cm
